# P28 Recalcitrant exuberant digital calcinosis cutis in a patient of CREST syndrome - A case report

**DOI:** 10.1093/rap/rkac067.028

**Published:** 2022-09-28

**Authors:** Yeshwanth Kempanna

**Affiliations:** Rheumatology, Northampton General Hospital, Northampton, United Kingdom

## Abstract

**Introduction/Background:**

Calcinosis cutis is dystrophic soft-tissue calcification associated with connective tissues diseases, especially scleroderma, systemic lupus erythematosus or dermatomyositis. It occurs in all subsets of scleroderma but is more prominent in patients with limited scleroderma (CREST syndrome) and in those with anticentromere antibody. Dystrophic calcinosis can be associated with severe pain, decreased mobility, increased risk of infection, and significantly decreased quality of life. We report our experience with a case of refractory calcinosis cutis in a patient with multiple comorbidities.

**Description/Method:**

We are presenting a 62-year-old lady diagnosed with Limited Cutaneous Sclerosis (CREST syndrome) with background of Giant Cell Arteritis, Osteoporosis, TIA and Hypothyroidism. Patient has had a history of onset of Raynaud’s at the age of 20 years. Around the age of 50, she started developing gastrointestinal features and subcutaneous nodules on her fingers. The painful nodules on her fingers were her predominant symptom. These nodules appeared as multiple hardened exuberant erythematous-whitish nodules, some having a chalky appearance, around her fingers. A clinical diagnosis of calcinosis cutis was made. Other features included sclerotic cutaneous findings on her face and fingers (sclerodactyly), prominent facial telangiectasia and esophageal reflux. In light of suspected CREST syndrome, patient was extensively investigated, and diagnosis was confirmed. Antinuclear and anti-centromere (anti-Th and anti-RNP) antibodies were positive. X-ray showed extensive calcinosis in the soft tissue of the fingers. Serum calcium and phosphorus levels were within the normal ranges. Pulmonary function test was normal. For her ongoing digital calcinosis cutis associated with severe pain and recurrent ulceration which was adversely impacting the quality of her life, she was started on low dose aspirin to alleviate her symptoms. This initially helped improve her symptoms, however later stopped responding. Then a trial of low dose Irbesartan at dose of 75 mg was given with which she found no relief. She was referred to higher center and a trail of Minocycline at dose of 50-100mg was given for over 3 months which proved to be of no benefit. Reports suggest bisphosphonates are helpful in Calcinosis cutis and though patient was Zoledronic acid for osteoporosis, her calcinosis cutis continued to grow and ulcerate. In view of recalcitrant nature of these lesions, patient has been referred to the plastic surgeons for surgical modality of treatment currently.

**Discussion/Results:**

Calcinosis cutis is a rare and chronic condition characterized by deposition of insoluble calcium salts in the skin and subcutaneous tissues. Dystrophic calcinosis is the most common type of calcinosis cutis and is seen in association with autoimmune connective tissue diseases. It is thought to occur as a result of chronic local tissue injury and is a common complication of systemic sclerosis especially the limited form (CREST syndrome: calcinosis, raynaud's phenomenon, esophageal dysmotility, sclerodactyly and telangiectasia), affecting approximately 25% of these patients. Calcinosis may develop at any time during the disease course and may predate the diagnosis of scleroderma, but typically occurs at least 10 years following diagnosis. Lesions occur on the hands and feet, with a high predilection for fingertips and areas of microtrauma. Raynaud’s and digital ulcers are risk factors for calcinosis in scleroderma, suggesting a role for vascular ischemia. These lesions might be associated with pain, soft tissue swelling, ulcers with toothpaste-like material discharging or deformities, which may lead to functional problems. Treatment of calcinosis cutis is tough and challenging as there is no gold standard treatment. Medical therapy for cutaneous calcinosis is limited and has variable benefits. Multiple treatment approaches with diltiazem, disodium etidronate, probenecid, colchicine, minocycline, low-dose warfarin, intralesional adrenal steroids, abatacept, rituximab, thalidomide have been explored, but no standard treatment has convincingly prevented or reduced calcinosis. Surgery is generally reserved for discrete lesions with problems of recurrent infection, severe pain, or functional impact Despite concerns about recurrence of lesion due to mechanical trauma at the operative site, surgical resection has been effective in treating calcinosis. Treatment with extracorporeal shock wave lithotripsy and carbon dioxide laser therapy have shown promise in a few cases and need further study.

**Key learning points/Conclusion:**

Pharmacological treatment of calcinosis cutis is difficult and a variety of drugs including bisphosphonates, intralesional corticosteroids, aluminium hydroxide, warfarin and diltiazem, have been tried with limited success. The local excision of painful or ulcerated nodule is the current existing therapeutic potion but local recurrence is common. The present case is of interest because it has features of Raynaud’s phenomenon, heart burn, telangiectasia and sclerodactyly with microscopic finding of calcinosis cutis (CREST syndrome). Patient with crest syndrome often have better prognosis than diffuse systemic sclerosis. Pathologist should be aware about various clinico-pathological categories associated with localized calcium deposition in dermis.

